# Toward Brain-Computer Interface motor rehabilitation for people with Multiple Sclerosis

**DOI:** 10.3389/fmed.2025.1661972

**Published:** 2026-01-05

**Authors:** Marc Sebastián-Romagosa, Woosang Cho, Rupert Ortner, Sebastian Sieghartsleitner, Michael Guger, Tim J. von Oertzen, Sven G. Meuth, Steven Laureys, Brendan Z. Allison, Christoph Guger

**Affiliations:** 1 g.tec Medical Engineering Spain SL, Barcelona, Spain; 2 g.tec Medical Engineering GmbH, Schiedlberg, Austria; 3 Department of Neurology, Pyhrn-Eisenwurzen Hospital Steyr, Steyr, Austria; 4 Medical Faculty of Johannes Kepler University Linz, Linz, Austria; 5 University Hospital Würzburg, Germany; 6 Universitätsklinikum Düsseldorf, Düsseldorf, Germany; 7 GIGA Consciousness Research Unit, University Liège, Antwerp, Belgium; 8 TRAINM, Antwerp, Belgium; 9 Department of Cognitive Science, University of California San Diego, La Jolla, CA, United States

**Keywords:** Brain-Computer Interface, Multiple Sclerosis, gait rehabilitation, EEG, motor imagery (MI), functional electrical simulation (FES), virtual reality, motor rehabilitation

## Abstract

**Background:**

Multiple Sclerosis (MS) is a chronic neurodegenerative disease in which the immune system attacks the myelin sheaths around nerves. People with MS (pwMS) often experience pain, fatigue, cognitive dysfunction, and reduced mobility. Today, MS is incurable, and treatments can at best slow the progression of the disease and manage symptoms. We conducted a preliminary, single-arm study using a motor-imagery brain–computer interface (MI-BCI) with functional electrical stimulation (FES) and virtual reality avatar targeting gait in pwMS.

**Methods:**

Twenty-six pwMS were enrolled; 24 completed 30 BCI sessions. Outcomes were assessed at Baseline, immediately post-treatment (Post1, week 13) and during follow-up (Post2, week 17; Post3, week 37). Change from baseline was analyzed using mixed models for repeated measures (with log-ratio models for skewed measures) and multiplicity control. This uncontrolled study is hypothesis-generating.

**Results:**

Patients treated with the BCI-based intervention obtained significant improvements that were largely maintained to 6 months after the therapy. The walking endurance, assessed by the 6-minute walking test (6MWT), increased by 37.3 m (95% CI 21.50–53.10) after the treatment (*p* < 0.001), exceeding the minimal clinically important difference (MCID). This improvement in the walking endurance was maintained during the following 6 months after the intervention. Mobility/speed improved: TUG and T25FW times decreased by −15.5% and −16.4% after the last BCI session (both *p* < 0.001), with benefits persisting after 6 months. Spasticity (MAS) declined by about 1 point, and patient-reported outcomes improved statistically and clinically (MSIS-29 10.18 points, MFIS 7.29 points). Pairwise post-visit contrasts were not significant, consistent with maintenance. Exploratory models found no consistent MS-subtype effect on 6MWT change and suggested larger gains with higher baseline EDSS. Two discontinuations were due to participant availability, not concerns with fatigue or safety.

**Conclusion:**

In this preliminary, single-arm study, a MI-BCI + FES system was associated with statistically significant, clinically meaningful gains in gait endurance, mobility/speed, spasticity, and patient-reported outcomes, sustained up to 6 months after the intervention.

## 1 Background

Multiple Sclerosis (MS) is an advanced neurologic disease that demyelinates neurons in the central nervous system. People with Multiple Sclerosis (pwMS) experience various symptoms such as impaired mobility (1), limited physical activity (2), and reduced quality of life (3). One of the most common symptoms after diagnosis is gait impairment (4). Walking impairment affects many aspects of a person’s life by limiting daily activity due to reduced walking endurance and increased dependence on caregivers. While disease-modifying drugs are available, behavioral and adjunctive rehabilitative approaches have emerged over the years as possible add-on treatments for pwMS. Exercise was historically discouraged because of concerns about exacerbating symptoms (5); however, accumulating evidence now supports its safety and benefit, leading to specific exercise recommendations for pwMS (6). Exercise-based interventions are associated with improvements in mobility, fatigue, and health-related quality of life (7–10).

In traditional rehabilitation approaches, patients are typically instructed to either move the affected limb or to imagine its movement, often with assistance from a physiotherapist (7). However, feedback is frequently delivered regardless of whether the patient is mentally engaged in the intended motor imagery (MI) task. For individuals unable to produce overt movement, it is difficult to objectively confirm whether they are actively engaging in MI and generating the corresponding neural activity. Synchronizing sensory feedback with intentional motor activity is a crucial element of effective motor rehabilitation (11–13). Neural pathways are reinforced when both presynaptic and postsynaptic neurons are activated simultaneously. If feedback is provided without concurrent MI, the necessary co-activation does not occur. This lack of synchronization between sensory input and motor intention may hinder neuroplastic changes near the damaged brain regions. Since Hebbian learning—the mechanism underlying experience-dependent neural reorganization—depends on such synchronized activity, unsynchronized feedback in conventional therapies can reduce treatment efficacy (14, 15). Therefore, the open-loop nature of these therapies may limit their potential to drive meaningful motor recovery.

Today, neurofeedback technology implemented through Brain-Computer Interfaces (BCIs) can provide an objective tool to measure MI, creating new possibilities for “closed-loop” feedback (15). Several review articles analyzed MI interventions in various neurological disorders (stroke, Parkinson’s disease, spinal cord injury, and amputation), underscoring the positive effects of mental training on motor performance (11, 16, 17) and highlighting changes in motor impairment favored by MI (18, 19). Although the evidence base is modest in MS, MI–based rehabilitation paired with musical and verbal cues has been associated with improvements in gait, fatigue, and quality of life in pwMS with low EDSS scores compared with no intervention or alternative therapies (20, 21). Rehabilitation using BCIs in pwMS remains underexplored. To our knowledge, a single interventional trial in pwMS—published in 2021 with seven participants—has evaluated BCI-based rehabilitation, reporting significant improvements in gait parameters after 24 sessions over 8 weeks (22).

A recurring practical question in the design of new BCI devices is which feedback modality best supports motor relearning in this population. Functional electrical stimulation (FES) is a widely used feedback modality in BCI-assisted rehabilitation. FES has also been used in walking improvement therapy over the last decades since Liberson et al. (23) first used it in 1961 for foot drop caused by stroke. It produces a muscle contraction in a paralyzed limb to regain motor control through electrical excitation of the innervating nerve. It is known that passive FES therapy can reduce muscle spasms and shorten the term of motor recovery in post-stroke patients (24). Evidence for pwMS is more limited but includes reports of functional gains with continuous passive motion or cycling paradigms in individuals with moderate to severe disability (25–29). Importantly, FES alone does not permit monitoring of MI-related neural activity. Visual feedback is another effective modality; action-observation, mirror therapy (MT) and virtual reality (VR)-based interventions have demonstrated functional benefits in acquired brain damage and Parkinson’s Disease (30–32), with emerging evidence suggesting potential applicability to pwMS (33, 34).

These considerations motivate the use of closed-loop approaches that couple MI detection with coherent feedback to maximize the rehabilitation potential for pwMS. Accordingly, this study explores the effectiveness of an MI-BCI-based system with FES feedback for gait rehabilitation in people with moderate to severe disability caused by Multiple Sclerosis.

## 2 Materials and methods

### 2.1 Participants and study design

The study was approved by the Ethikkommission des Landes Oberösterreich (number: 1256/2021) and the Bundesamt für Sicherheit im Gesundheitswesen (reference number: 100431456) in Austria. Each participant provided written informed consent before the pre-assessment. No adverse events were reported during the entire study period.

The following inclusion criteria were applied to all participants: Clinician-confirmed diagnosis of Multiple Sclerosis of any phenotype; age at least 18 years; able to understand written and spoken instructions; neurological restrictions of the lower extremities preventing the participant from activities of daily life; stable neurological status; willing to participate in the study; able to understand and sign the informed consent; available for consecutive therapy sessions; Expanded Disability Status Scale (EDSS) ≤ 6.5; able to provide detailed MS diagnostic information, including brain images; able to perform 6-minute walk test (6MWT) with or without walking aid; no adjustment of disease-modifying medication or medication that affects gait performance and spasticity within the past 2 months; no participation in any physical activity program in the last 2 months.

Participants were excluded from the study if they met any of the following conditions: pregnancy; joint abnormalities such as ossification, contractures, or stiffness in the wrist or ankle; presence of metal objects (e.g., jewelry, piercings, buckles, surgical staples) in the stimulation area; severe visual or auditory impairments that would interfere with task performance; intolerance to surface electrical stimulation; implanted medical devices including pacemakers; metallic implants in the limbs that could interfere with FES; cerebellar lesions; diagnosis of a MS relapse within the past 3 months; prior history of stroke; elevated intracranial pressure; significant hemineglect; uncontrolled epilepsy or seizure disorders; current use of anesthesia or sedative medications; fractures or injuries in the targeted limbs; severe respiratory, infectious, renal, hepatic, or cardiac conditions; pronounced Pusher syndrome; poor circulation in the stimulated extremities; inability to sit unassisted for approximately 60 min; sensory deficits impairing pain perception or proprioception; peripheral nervous system disorders affecting the limbs; recent botulinum toxin treatment of the affected lower limb; significant cognitive impairment or major psychiatric disorders (e.g., severe depression, psychosis, dementia); substance dependence (e.g., alcohol); or any neurological condition other than MS.

Each participant underwent 2.5 months of BCI-assisted motor imagery (MI) training, consisting of three sessions per week for a total of 30 sessions. Functional assessments were conducted at five time points: two before the intervention (Pre1 and Pre2) and three afterward (Post1, Post2, and Post3). These evaluations were carried out by two independent certified physiotherapists and subsequently reviewed by the research team. Pre1 and Pre2 were administered approximately 1 month and a few days prior to the start of therapy, respectively. The post-intervention assessments took place a few days after completing the therapy (Post1−week 13), then at 1 month (Post2−week 17), and again at 6 months (Post3−week 37) following the final session.

#### 2.1.1 Distribution of the BCI sessions

The 30 treatment sessions were divided into 2–3 sessions per week. The BCI system has two different training paradigms: “Right Foot - Left Hand” (RF-LH) and “Right Hand - Left Foot” (RH-LF). The RF-LH paradigm was used in the even sessions, while the RH-LF paradigm was used in the odd sessions. All patients completed 15 sessions of each paradigm in total. This was the most effective way to treat all extremities while maintaining adequate spatial mapping of the motor cortex with EEG and movement classification accuracy.

### 2.2 Sample size calculation

The sample size calculation (35) considered three parameters; α = 0.05, power = 0.8 and the substantial meaningful change in gait endurance of pwMS = 29.2 m (36). The resulting value of this calculation was 29 participants with a drop-out rate of 20%, or 24 participants completing the study.

### 2.3 Functional assessment

A series of functional and behavioral scales were administered in pre- and post-assessments. The primary outcome measure was gait endurance assessed by the 6-minute walk test (6MWT). This test assesses the distance that a person walks in 6 min. Gait was also evaluated with the Timed 25-Foot Walk (T25FW). The patient is directed to walk 25 feet as quickly and safely as possible from one marked end to the other. The time is calculated from the initiation of the person instructed to begin until the patient has reached the 25-foot mark. Scoring for the T25FW is the average of two trials. The second trial is administered immediately after the first trial by asking the person to walk the same distance.

Coordination and balance were assessed by the Timed Up and Go test (TUG). This scale consists of standing up, walking 3 m, turning around, walking back 3 m, and sitting down. We used the Modified Ashworth Scale (MAS) to assess spasticity, in which low scores reflect less spasticity (37). Spasticity was assessed in the left and right ankles.

Participants were permitted to use their customary assistive devices (e.g., cane, walker, ankle–foot orthosis) during the gait testing. The same device and settings were required across all assessment sessions to ensure comparability.

Finally, we used two specific scales to evaluate MS. The 29-item Multiple Sclerosis Impact Scale (MSIS-29) is a disease-specific measure that examines the impact of MS on physical and psychological functioning. It is composed of two scales: the physical impact subscale (PHYS) and the psychological impact subscale. Each item is scored on a five-point Likert scale, 1- Not at all, 2- A little, 3- Moderately, 4- Quite a bit, 5- Extremely. The second scale specific for MS is the 21-item Modified Fatigue Impact Scale (MFIS). This measure examines the impact of fatigue on MS patients. Each item is scored on a five-point Likert scale, 0- Never, 1- Rarely, 2- Sometimes, 3- Often, 4- Almost Always.

### 2.4 BCI system description

**FIGURE 1:**
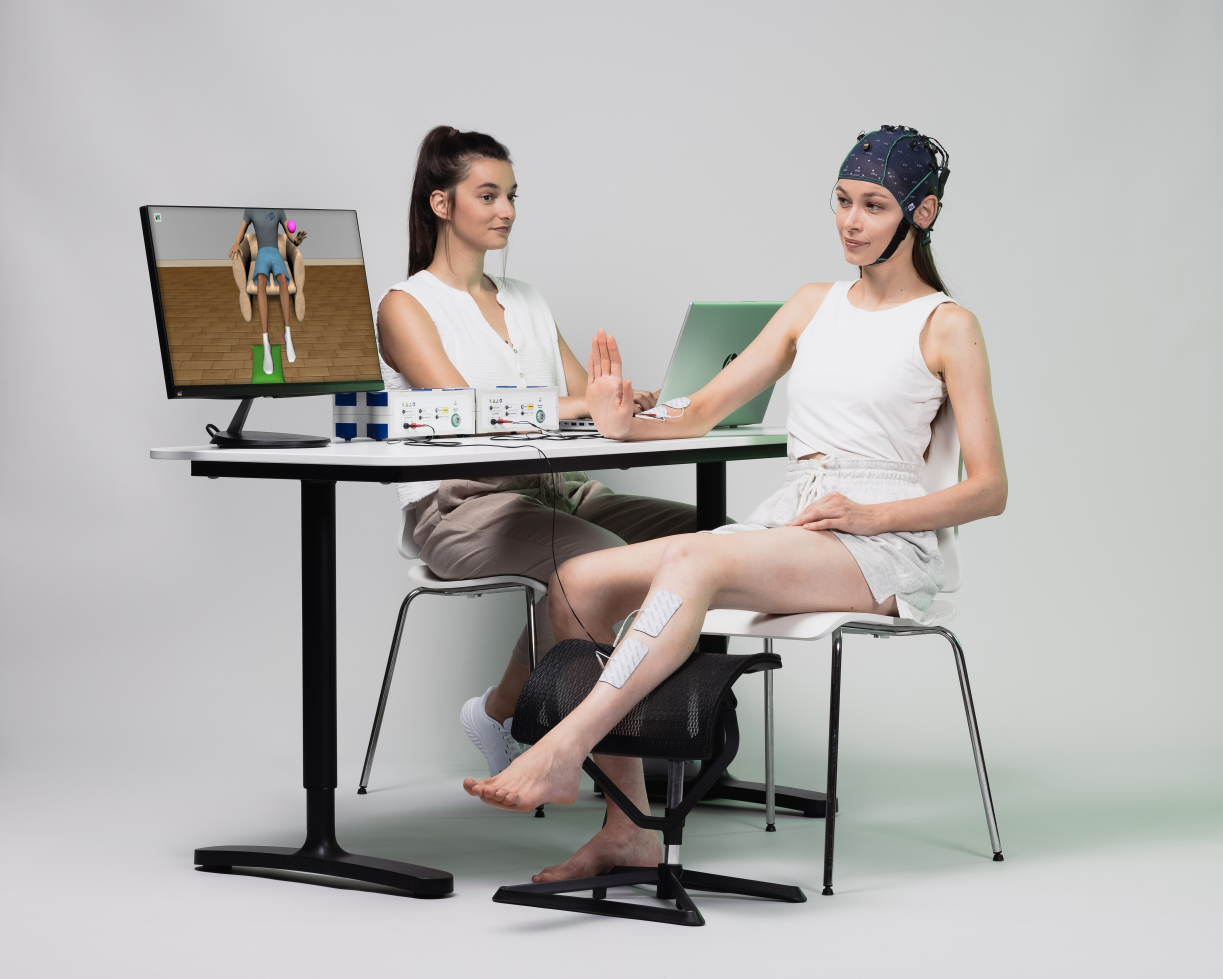
The photograph illustrates the key components of the Brain–Computer Interface (BCI) system used in this study. It includes a monitor displaying an avatar that guides the patient and provides visual feedback. Brain activity is recorded in real time via an EEG system, while a functional electrical stimulation (FES) device—shown on the table—is connected to electrodes placed on the right arm and left leg, representing a Right Hand–Left Ankle (RH-LA) session. When the BCI detects motor imagery of the corresponding limb, the avatar performs the intended movement, and the FES delivers stimulation to induce that movement physically.

The BCI system used in this clinical trial is called recoveriX PRO from g.tec medical engineering GmbH. The main components of the system are: the EEG cap with 16 active electrodes (g.Nautilus PRO, g.tec medical engineering GmbH, Austria); two functional electrical stimulators (g.Estim FES, g.tec medical engineering GmbH, Austria); the VR monitor where the patient can see the movements of the avatar triggered by the MI performance; and the computer where the software is installed, the different components are integrated and the brain signals are processed. See Figure 1.

The positions of the EEG electrodes and the specifications regarding the use of the FES device are described in a previous publication by the same research group (38).

Participants were guided to perform motor imagery tasks involving wrist or ankle dorsiflexion, as directed by the system. Each session consisted of three runs, with 40 trials per limb type (wrist or ankle), resulting in a total of 240 motor imagery trials per session. The entire session, including setup and cleanup, lasted approximately 1 h.

**FIGURE 2:**
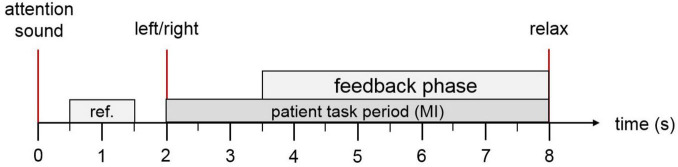
Trial overview: Each trial begins with an auditory cue to capture the patient’s attention. At the 2-s mark, an arrow appears on the screen indicating whether the patient should imagine movement of the ankle or hand, accompanied by a verbal instruction in the patient’s native language. If the motor imagery is correctly detected, both the virtual avatar and the functional electrical stimulation (FES) system are activated to provide visual and physical feedback. At second 8, a relaxation cue signals the end of the trial.

Motor imagery (MI) tasks were delivered in a pseudo-randomized sequence, with variable intervals between trials. Figure 2 illustrates the temporal structure of each trial. At trial onset, an attention beep signaled the participant to prepare. After 2 s, an animated arrow appeared in the avatar display, indicating the target limb for the MI task, accompanied by a verbal instruction specifying whether to imagine movement of the left or right side. In RF-LH sessions, these cues corresponded to right foot and left-hand movements; the opposite applied during RH-LF sessions. If the BCI system successfully detected the intended MI, both the avatar and FES were activated to provide feedback. If MI was not detected, feedback remained inactive. The feedback loop was refreshed five times per second to ensure timely responsiveness.

The signal processing pipeline and the calculation of the MI accuracy parameter are explained in previous publications (38–40).

### 2.5 Statistical analysis methods

Statistical analyses were conducted using MATLAB R2024b and RStudio. For each outcome variable, the baseline value was defined as the average of the two pre-intervention assessments [Baseline = (Pre1 + Pre2)/2], while the post-intervention values corresponded to the assessment conducted after the 30 therapy sessions (Post1−W13), 1 month after the therapy (Post2−W17) and 6 months after the therapy (Post3−W37). Normality of the data distribution was evaluated using the Shapiro-Wilk test, and a significance level of α = 0.05 was applied throughout the analysis. The selection of statistical tests was based on data distribution, homogeneity of variance (as assessed by Levene’s or Brown-Forsythe test), and sample size. Descriptive statistics are presented as mean ± standard deviation (SD) or median with interquartile range (IQR: 25th–75th percentile), depending on the data characteristics.

To analyze treatment-related change while accounting for within-subject correlation, mixed models for repeated measures (MMRM) were implemented. For variables that satisfied normality assumptions [e.g., 6-minute walk test (6MWT), MSIS-29, MFIS], the dependent variable was the change from baseline, with visit (Post1–Post3) as a categorical fixed effect, and baseline value (centered) included as a covariate. For time-based outcomes with significant positive skew [Timed Up and Go Test (TUG) and Timed 25-Foot Walk (T25FW)], data were log-transformed, and analyses were performed on the log-ratio of Post/Baseline values, enabling interpretation of results as geometric mean ratios (GMRs) and percentage changes.

All models included a random intercept for subject to account for inter-individual variability. The within-subject covariance structure was selected based on the Akaike Information Criterion (AIC) from tested structures {compound symmetry, autoregressive [AR(1)], and unstructured}. Model parameters were estimated using restricted maximum likelihood (REML), and Kenward–Roger or Satterthwaite approximations were applied to adjust degrees of freedom.

Primary comparisons evaluated Post1, Post2, and Post3 versus baseline. Multiplicity across the three post-treatment visits within each scale was controlled using the Holm step-down procedure, maintaining the family-wise error rate (FWER) at 0.05. Exploratory pairwise comparisons among post-treatment visits were conducted using Tukey–Kramer adjusted *p*-values and confidence intervals. All estimated marginal means (EMMs) were extracted using the emmeans package, and back-transformed when applicable.

Model assumptions were evaluated via residual diagnostics (normality, homoscedasticity, influential cases). Sensitivity analyses using non-parametric paired Wilcoxon tests were performed for Baseline vs. Post3 comparisons to support robustness of findings. Statistical significance was set at *p* < 0.05 (two-sided).

#### 2.5.1 Handling missing data

The EDSS score was missing for two patients (2/24, 8.3%). We performed multiple imputation using predictive mean matching (PMM; *m* = 50, donors = 15) with Sex, SMWT, T25WT, TUG, and MSIS as predictors. For the baseline table, imputed EDSS values were rounded to 0.5-point increments, and descriptive statistics were averaged across imputations. The MS subtype was not imputed and remained “Unknown” when undocumented.

#### 2.5.2 Statistical analysis of functional change

For each outcome—Δ6MWT, ΔTUG, and ΔT25WT—change scores were computed as Post1-Baseline (more positive = improvement for 6MWT; more negative = improvement for TUG/T25WT). We fitted linear regression models with change as the dependent variable and Sex (factor), MS subtype (factor: RRMS, PPMS, SPMS, Unknown), and EDSS (centered; EDSS_c) as predictors. Global effects were evaluated with Type-III ANOVA (to account for unbalanced groups). As a sensitivity analysis, we reported heteroskedasticity-robust standard errors (HC3) for regression coefficients. We also summarized adjusted marginal means by MS subtype and simple slopes of EDSS within subtypes using emmeans/emtrends. Model assumptions (linearity, normality of residuals, homoscedasticity, influential points) were checked with standard diagnostics; no transformations were applied. Two-sided α = 0.05 defined statistical significance.

Analyses were conducted in R (version 2025.09.0 Build 387) using the car, emmeans, sandwich, and lmtest packages.

## 3 Results

### 3.1 Patients’ baselines

Twenty-six people with Multiple Sclerosis (pwMS) were recruited; two discontinued due to difficulties attending treatment sessions (drop-out < 10%). Analyses include the 24 completers (six males, 18 females; mean age = 54.06 years, SD = 9.54; median time since diagnosis = 17.4 years). Regarding MS subtype, 9 (37.5%) had relapsing-remitting MS (RRMS), 8 (33.3%) had primary-progressive MS (PPMS), 4 (16.7%) had secondary-progressive MS (SPMS), and 3 (12.5%) had unknown subtype. Regarding disability, the Expanded Disability Status Scale (EDSS) distribution was: 3.5 (*n* = 2), 4.0 (*n* = 4), 4.5 (*n* = 6), 5.0 (*n* = 1), 5.5 (*n* = 2), 6.0 (*n* = 3), and 6.5 (*n* = 6).

### 3.2 Functional improvement after BCI therapy

This section presents the differences observed between baseline and the post-intervention assessments (Post1−W13, Post2−W17, and Post3−W37) across various functional measures, as detailed in Table 1. All evaluations were conducted in a standardized sequence at each assessment point to ensure consistency.

**TABLE 1:** Summary of the results in the functional scales across different assessments.

Scale name	Units	*n*	p_SW	w_SW	Mean	SD	Median	Q1	Q3
**Baseline**									
MAS	Score	24	0.033	0.909	3.14	2.66	2.75	1.38	4.31
MFIS	Score	24	0.21	0.945	35.52	13.6	37.25	25.25	48.88
MSIS29	Score	24	0.746	0.973	73.98	16.88	73.25	61	82.25
SMWT	Distance (m)	24	0.198	0.944	233.67	141.53	205.06	122.92	321.99
T25WT	Time (s)	24	<0.001	0.758	13.4	11.25	9.67	6.29	15.33
TUG	Time (s)	24	<0.001	0.797	25.02	17.74	18.77	13.04	31.44
**Post1**									
MAS	Score	23	0.001	0.819	2.46	2.75	2	0	3
MFIS	Score	24	0.86	0.978	28.29	16.02	29.5	17	38
MSIS29	Score	24	0.141	0.937	63.83	19.24	59.5	50.75	76
SMWT	Distance (m)	24	0.265	0.95	270.96	144.08	263.4	133.8	355.53
T25WT	Time (s)	24	0.001	0.829	10.26	6.58	8.04	5.59	12.26
TUG	Time (s)	24	0.001	0.824	20.48	13.05	16.97	10.91	25.35
**Post2**									
MAS	Score	22	0.002	0.83	1.95	2.19	1	0	2.88
MFIS	Score	22	0.234	0.944	30.23	14.82	28	24	36
MSIS29	Score	22	0.602	0.965	63.23	18.49	62	51.25	73
SMWT	Distance (m)	22	0.111	0.928	255.83	147.45	223.7	133.46	317.13
T25WT	Time (s)	22	0.001	0.813	10.45	7.01	8.21	5.97	13.23
TUG	Time (s)	22	0.001	0.811	19.6	12.71	15.58	11.13	24.38
**Post3**									
MAS	Score	17	<0.001	0.744	1.76	2.38	1	0	2.5
MFIS	Score	16	0.095	0.905	30.88	13.31	33.5	29	39.25
MSIS29	Score	16	0.719	0.963	62.81	18.09	65	52.75	71.25
SMWT	Distance (m)	17	0.336	0.941	278.35	148.45	260	198	404.3
T25WT	Time (s)	16	<0.001	0.714	12.3	11.32	8.26	5.19	12.7
TUG	Time (s)	16	<0.001	0.729	22.02	18.08	13.52	11.06	22.26

n, number of participants who completed the assessment; p_SW, *p*-value for the Shapiro–Wilk test; w_SW, Shapiro–Wilk W statistic; Q1, 25th percentile (first quartile); Q3, 75th percentile (third quartile); TUG, Timed Up and Go; 6MWT, 6-minute walk test; MSIS-29, Multiple Sclerosis Impact Scale-29; MFIS, Modified Fatigue Impact Scale; MAS, Modified Ashworth Scale.

#### 3.2.1 Functional outcome measures: 6MWT, T25FW, TUG, and MAS

##### 3.2.1.1 6 -minute walk test (6MWT)

**FIGURE 3:**
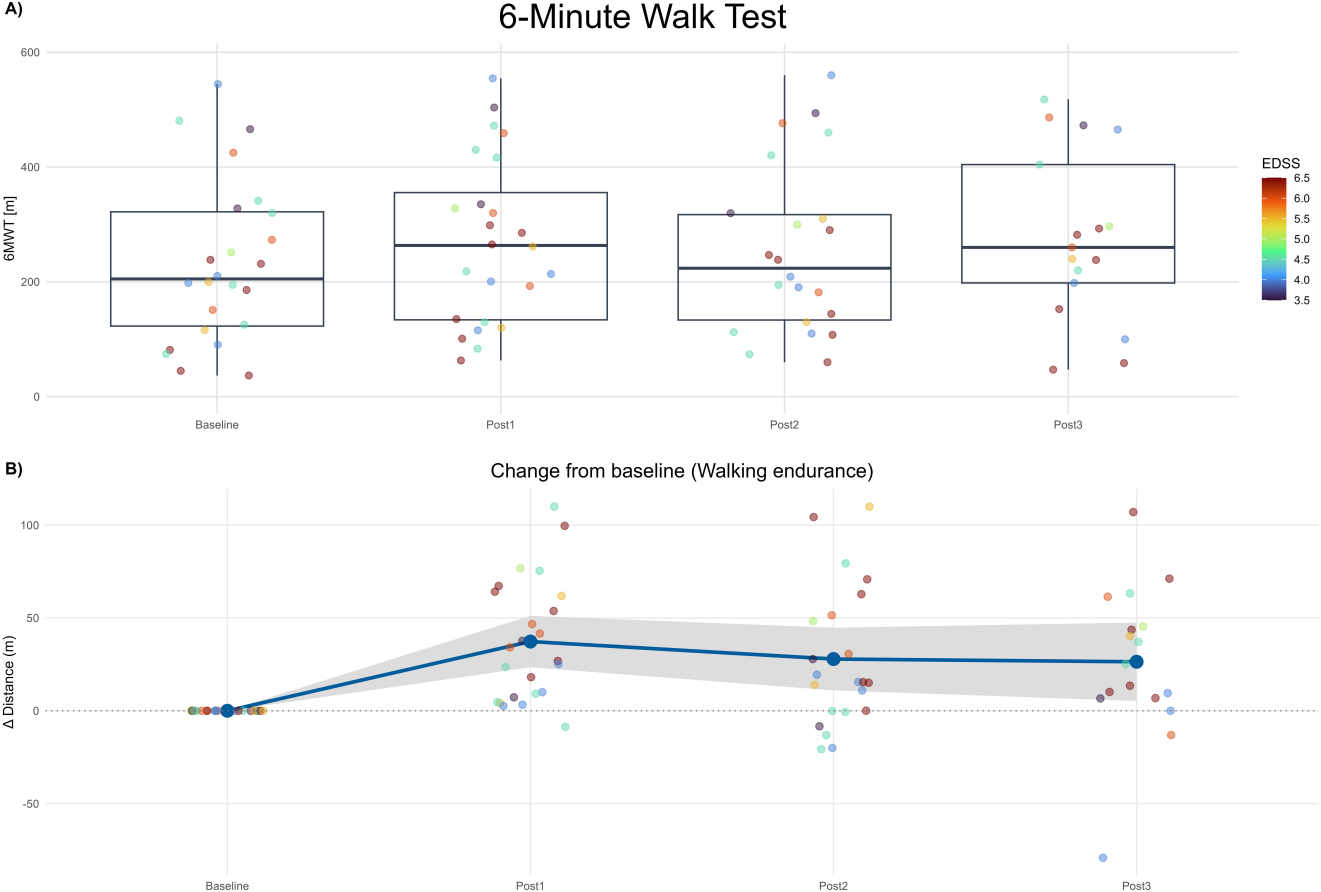
6-minute walk test (6MWT). (A) Boxplots of 6MWT distance (meters) at Baseline and each post-assessment (Post1–Post3). Points show individual participants; point color encodes EDSS (imputed). (B) Change from baseline in 6MWT distance (Δ meters) across post-assessments. The blue line depicts the mean change and the gray band the 95% confidence interval. Positive values indicate improvement.

Normality testing by Shapiro–Wilk indicated no significant deviation from normality. Therefore, an MMRM on change from baseline was appropriate. Adjusted least-square mean (LS-mean) increases in walking distance were + 37.30 m at Post1 (SE 7.62; 95% CI 21.50–53.10; Holm-adjusted *p* < 0.001), + 29.60 m at Post2 (SE 7.78; 95% CI 13.50–45.70; *p* = 0.002), and +21.40 m at Post3 (SE 8.42; 95% CI 4.00–38.80; *p* = 0.018). Pairwise Tukey adjustments between Post1−Post2, Post1−Post3, and Post2−Post3 showed no significant differences (*p* ≥ 0.126), implying maintenance of improved walking endurance over the 6-month follow-up. Given recommended MCID values (19.7 m improvement, deterioration threshold 7.2 m) in MS populations (41), the observed improvements are not only statistically significant but clinically meaningful and sustained. See Figure 3.

##### 3.2.1.2 Timed Up and Go Test (TUG)

The values of TUG scale were not normally distributed; therefore, the analysis of change from baseline was performed using a mixed-model for repeated measures (MMRM) on the log-transformed ratio of post-treatment to baseline TUG times. The geometric mean ratios (GMR; Post/Baseline) were 0.845 at Post1 (−15.5%; 95% CI 0.808–0.884; Holm-adjusted *p* < 0.001), 0.791 at Post2 (−20.9%; 95% CI 0.736–0.850; *p* < 0.001), and 0.825 at Post3 (−17.5%; 95% CI 0.742–0.918; *p* = 0.001). All post-treatment visits demonstrated statistically significant reductions in TUG time relative to baseline, indicating a robust improvement in mobility and functional ambulation.

**FIGURE 4:**
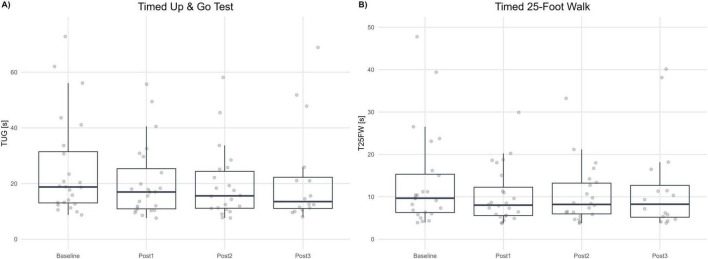
Gait speed and mobility tests. **(A)** Timed Up and Go (TUG, seconds) at Baseline and each post-assessment (Post1–Post3). **(B)** Timed 25-Foot Walk (T25FW, seconds) at Baseline and each post-assessment. Boxplots summarize distributions; dots represent individual participants. Lower values indicate better performance.

Pairwise Tukey-adjusted comparisons between post-treatment visits (Post1−Post2, Post1−Post3, Post2−Post3) revealed no significant differences (*p* ≥ 0.158), suggesting that the reduction in TUG time achieved immediately after therapy was sustained over the 6-month follow-up period. This maintenance of effect indicates persistence of the functional gains rather than transient or short-term benefits. See Figure 4A.

##### 3.2.1.3 Timed 25-Foot Walk (T25FW)

The T25FW values were not normally distributed. Thus, log-transformed data and an MMRM on log-ratio Post/Baseline were implemented. Geometric mean ratios (GMR) were 0.873 (−16.4%; 95% CI 0.77–0.90; *p* < 0.001) at Post1, 0.823 (−17.7%; 95% CI 0.753–0.814; *p* < 0.001) at Post2 and 0.934 (−6.6%; 95% CI 0.814–1.07; *p* = 0.309) at Post3. Tukey-adjusted pairwise contrasts between post-visits were not statistically significant (*p* ≥ 0.107), indicating functional gains in gait speed remained consistent. See Figure 4B.

##### 3.2.1.4 Modified Ashworth Scale (MAS)

Modified Ashworth Scale scores displayed non-normal distribution across visits (all Shapiro–Wilk *p* < 0.05). Therefore, a mixed-model for repeated measures (MMRM) was applied to change from baseline, coded such that positive values indicate improvement (lower MAS = reduced spasticity). Adjusted LS-mean improvements were +0.65 points at Post1 (SE 0.27; 95% CI 0.09–1.20; *p* = 0.024), +1.29 points at Post2 (SE 0.21; 95% CI 0.86–1.73; *p* < 0.001), and +1.06 points at Post3 (SE 0.21; 95% CI 0.62–1.50; *p* < 0.001; Holm-adjusted). These effects were supported by a log-transformed sensitivity analysis, with geometric mean ratios (MAS + 1) vs. baseline of 0.78 at Post1 (95% CI 0.67–0.92; *p* = 0.005), 0.66 at Post2 (95% CI 0.54–0.80; *p* < 0.001), and 0.72 at Post3 (95% CI 0.59–0.88; *p* = 0.005), confirming a clinically meaningful reduction in spasticity.

Paired non-parametric tests further supported improvements (Wilcoxon Post1: *p* = 0.004; Post2: *p* < 0.001; Post3: *p* = 0.004). Clinically, 39% of participants at Post1 (95% CI 22%–59%), 59% at Post2 (95% CI 39%–77%), and 53% at Post3 (95% CI 31%–74%) achieved a ≥ 1-point reduction in MAS score. While the MMRM showed the largest mean improvement at Post2, Tukey-adjusted comparisons among post-treatment visits identified no significant differences between Post2 and Post3, indicating maintenance of therapeutic effect through 3 months of follow-up.

#### 3.2.2 Impact of Multiple Sclerosis on activities for daily living: MSIS-29 and MFIS

##### 3.2.2.1 Multiple Sclerosis Impact Scale (MSIS29)

**FIGURE 5:**
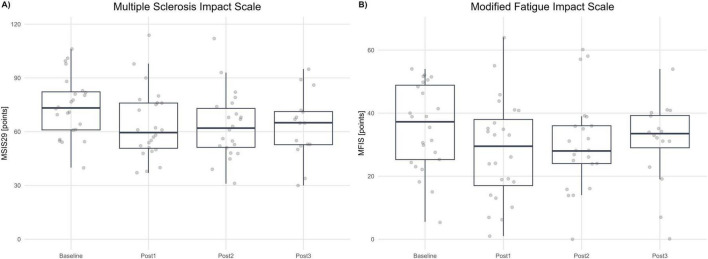
Patient-reported outcomes. **(A)** Multiple Sclerosis Impact Scale (MSIS-29, points) at Baseline and each post-assessment (Post1–Post3). **(B)** Modified Fatigue Impact Scale (MFIS, points) at Baseline and each post-assessment. Boxplots summarize distributions; dots represent individual participants. Higher scores indicate greater impact/fatigue (worse status).

Shapiro–Wilk testing supported approximate normality across visits; therefore, MSIS-29 was analyzed with an MMRM on change from baseline, coding positive values as improvement (lower raw scores = better status). Adjusted LS-mean improvements were +10.18 points at Post1 (SE 2.35; 95% CI 5.31–15.05; *p* < 0.001), +11.84 points at Post2 (SE 2.17; 95% CI 7.33–16.34; *p* < 0.001), and +10.42 points at Post3 (SE 2.45; 95% CI 5.33–15.51; *p* < 0.001). Tukey-adjusted pairwise comparisons among post-treatment visits were not significant (Post1−Post2: Δ = −1.65, 95% CI −5.96 to 2.65, *p* = 0.619; Post1−Post3: Δ = −0.23, 95% CI −4.28 to 3.81, *p* = 0.989; Post2−Post3: Δ = 1.42, 95% CI −3.15 to 5.99, *p* = 0.730), indicating maintenance of effect over follow-up. The magnitude of improvement (≈10–12 points) exceeds commonly cited MCID thresholds for the MSIS-29 physical impact domain, supporting clinically meaningful and sustained reductions in perceived disease impact. See Figure 5A.

##### 3.2.2.2 Modified Fatigue Impact Scale (MFIS)

Shapiro–Wilk testing indicated approximate normality across visits (with a borderline result at Post1); residual diagnostics from the MMRM were acceptable. MFIS was analyzed as change from baseline, coded so that positive values indicate improvement (lower raw MFIS = less fatigue impact). Adjusted LS-mean improvements were +7.29 points at Post1 (SE 2.09; 95% CI 2.96–11.61; *p* = 0.006), + 6.38 points at Post2 (SE 1.96; 95% CI 2.31–10.45; *p* = 0.007), and +5.27 points at Post3 (SE 2.66; 95% CI −0.24 to 10.78; *p* = 0.060; Holm-adjusted). Thus, improvements versus baseline were statistically significant at Post1 and Post2, with a consistent magnitude at Post3 that did not reach statistical significance after multiplicity correction. Given that published MCID estimates for MFIS are around four points, the mean improvements at Post1 and Post2 exceed this threshold, and the point estimate at Post3 is directionally consistent with a clinically meaningful reduction in fatigue impact, albeit with wider uncertainty. See Figure 5B.

Tukey-adjusted pairwise comparisons among post-treatment visits were not significant (Post1−Post2: Δ = 0.91, 95% CI −2.41 to 4.23, *p* = 0.783; Post1−Post3: Δ = 2.01, 95% CI −3.39 to 7.42, *p* = 0.638; Post2−Post3: Δ = 1.11, 95% CI −4.12 to 6.33, *p* = 0.864), indicating no evidence of differences between post visits and supporting a maintenance of effect across follow-up. Consistent with the MMRM results versus baseline (Post1: +7.29, *p* = 0.006; Post2: +6.38, *p* = 0.007; Post3: +5.27, *p* = 0.060), improvements were significant at Post1 and Post2, and directionally maintained at Post3 with wider uncertainty.

#### 3.2.3 Functional improvement based on the MS type and EDSS

We examined whether MS subtype and baseline disability (centered EDSS; EDSS_c) were related to post-therapy change in function. For each outcome (Δ6MWT, ΔTUG, ΔT25WT), we fitted linear models adjusted for sex, MS subtype (RRMS, PPMS, SPMS, Unknown), and EDSS_c.

##### 3.2.3.1 Change in walking endurance (Δ6MWT)

The model did not reach significance overall [F(5,18) = 1.43, *p* = 0.262; adjusted *R*^2^ = 0.085]. Neither sex nor MS subtype contributed meaningfully (Type-III: Sex, *F* = 0.71, *p* = 0.411; MS, *F* = 0.96, *p* = 0.432]. Higher EDSS_c showed a trend toward greater gains in 6MWT distance (β =+11.0 m per EDSS point; SE = 5.71; robust *t* = 1.93; *p* = 0.070; conventional *p* = 0.091). Adjusted mean Δ6MWT (95% CI) was 36.2 m (11.6–60.8) for PPMS, 31.3 m (8.8–53.9) for RRMS, 54.0 m (19.7–88.3) for SPMS, and 61.6 m (20.3–102.8) for Unknown; pairwise contrasts were not significant. Simple slopes for EDSS_c were similar across subtypes (≈+11 m; 95% CI −2.0 to 23.9).

##### 3.2.3.2 Change in mobility and coordination (ΔTUG)

For ΔTUG, the model explained a moderate amount of variance [F(5,18) = 2.84, *p* = 0.046; adjusted *R*^2^ = 0.285]. MS subtype showed a trend (*F* = 2.76, *p* = 0.072) and EDSS_c was borderline (*F* = 3.63, *p* = 0.073); sex was not associated (*F* = 0.49, *p* = 0.492). Relative to PPMS, SPMS displayed larger improvements (β = −7.50 s; SE = 2.70; *p* = 0.013), whereas RRMS and Unknown did not differ (both *p* > 0.49). Greater baseline disability tended to relate to larger improvements (EDSS_c β = −1.64 s per point; SE = 0.86; *p* = 0.073). With HC3 robust SEs, the pattern persisted but attenuated (SPMS *p* = 0.064; EDSS_c *p* = 0.148). Adjusted mean ΔTUG was −2.31 s (−5.74 to 1.12) for PPMS, −3.81 s (−6.96 to −0.66) for RRMS, −9.81 s (−14.59 to −5.02) for SPMS, and −2.71 s (−8.47 to 3.04) for Unknown, indicating the largest average improvement in SPMS.

##### 3.2.3.3 Change in walking speed (ΔT25WT)

Results for ΔT25WT mirrored those above but were stronger: the model was significant [F(5,18) = 4.00, *p* = 0.013; adjusted *R*^2^ = 0.395]. Both MS subtype (*F* = 3.89, *p* = 0.026) and EDSS_c (*F* = 5.36, *p* = 0.033) contributed, while sex did not (*p* = 0.897). SPMS improved more than PPMS (β = −9.75 s; SE = 3.04; *p* = 0.0049); RRMS and Unknown were not different (both *p* ≥ 0.56). Higher EDSS_c was associated with greater improvement (β = −2.24 s per point; SE = 0.97; *p* = 0.033). Using robust SEs, effects were directionally consistent but weaker (SPMS *p* = 0.147; EDSS_c *p* = 0.069). Adjusted mean ΔT25WT was −0.93 s (−4.79 to 2.93) for PPMS, −2.33 s (−5.87 to 1.22) for RRMS, −10.68 s (−16.06 to −5.30) for SPMS, and −0.75 s (−7.22 to 5.71) for Unknown.

### 3.3 BCI performance

**FIGURE 6:**
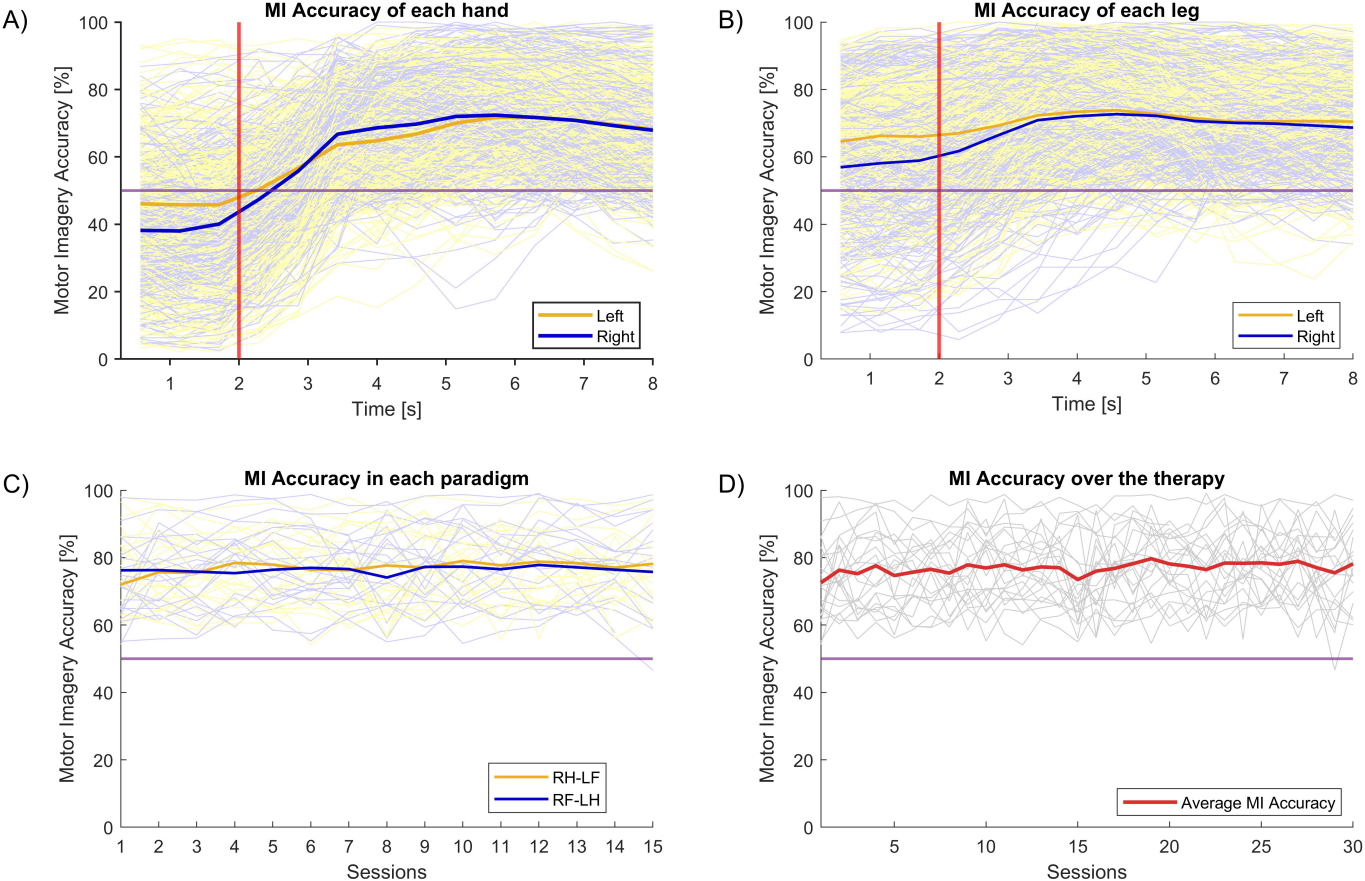
Motor Imagery Accuracy (MIAc). The horizontal purple line indicates the chance level. In panel **(A–C)**, the dim blue and orange lines indicate MIAc in each of two different classes for individual participants, while the solid lines reflect their averages. MIAc was similar for left vs. right classification in the hand **(A)** and leg **(B)**. **(C)** Shows that MIAc was similar for the two types of MI paradigm in this study across sessions. The dim gray lines in panel **(D)** indicates the individual subjects’ MIAc, while the solid red line is their average. **(D)** Shows that MIAc did not change substantially across sessions.

Brain–Computer Interface performance was assessed using Motor Imagery Accuracy (MIAc). This parameter is directly related to the physiological activity the motor cortex during a MI task. After the treatment session, the BCI device provides the MIAc parameter for each hand (MIAc_Right, MIAc_Left), and the average of both hands (MIAc_mean). Figure 6 summarizes the BCI performance.

Figure 6A shows the MIAc of each hand during the MI task. As explained in Figure 2, each MI task lasts for 8 seconds. The first 2 s are a resting period, after which the subject is cued to begin imagining movement and continue imagining movement for 6 s. The MIAc is calculated every 0.5 s. The thick lines of this plot are the mean MIAc of each hand.

Figure 6B shows the MIAc of the imagined leg movements. There is no significant difference in the MIAc between hands versus legs. The mean MIAc for the hands were: 68.59% (SD = 2.98) and 69.91% (SD = 1.96) in the left and right hands, respectively. The mean MIAc for the ankles were: 71.75% (SD = 1.34) and 70.71% (SD = 1.38) for the left and right ankles, respectively.

Figure 6C shows the MIAc of both hands for each task paradigm (RH-LF and RF-LH). All participants performed 15 sessions of each paradigm (30 sessions in total). There is no statistical difference between the MIAcs of these paradigms.

Finally, Figure 6D shows the average MIAc for all sessions. There are no statistical differences in the MIAc across sessions.

#### 3.3.1 BCI feedback dosage

The total feedback time was calculated using the raw data from each therapy session. We calculated the total functional electrical simulation time delivered to each limb and the time that the avatar was moving to recreate the MI task. The mean stimulation time per session is reported in minutes and (95% confidence interval).

The mean stimulation time per session for the left side was Ankle 6.88 min (6.77–6.97), Hand 7.07 min (6.97–7.17). The mean stimulation time per session for the right side was Ankle 6.80 min (6.72–6.89), Hand 7.10 min (7.00–7.21).

The stimulation time is directly related to the classifier’s performance for assessing the motor imagination. Therefore, there is a direct relationship between the stimulation time and the MIAc.

## 4 Discussion

The objective of this study was to evaluate the clinical effectiveness and safety of a Brain–Computer Interface (BCI) program specifically targeting gait rehabilitation in people with Multiple Sclerosis (pwMS). Of the 26 participants enrolled, two discontinued because of difficulties attending treatment sessions; the remaining 24 completed 30 BCI sessions each. Outcomes were assessed up to 6 months after the final session, enabling evaluation of both immediate and later effects. Participants demonstrated clinically meaningful improvements in walking endurance, gait speed, and mobility, alongside reductions in patient-reported disease impact following the BCI intervention.

### 4.1 Functional outcomes

Exercise-based rehabilitation is well-established for improving walking speed and endurance in pwMS; however, fatigue often limits participation, enthusiasm, and therapeutic impact. A distinctive feature of the present BCI paradigm is that training occurred in a seated position, minimizing exertional load while engaging task-relevant neural circuits via motor imagery and closed-loop feedback. This design aims to preserve clinical effectiveness while enhancing feasibility and safety in patients with activity-induced fatigability. Recent survey work in pwMS reports a strong interest in BCIs with a clear preference for non-invasive systems and highlights mobility support and fatigue as salient user priorities for at-home translation (42). These user-centered findings reinforce the clinical relevance of our seated, non-invasive EEG MI-BCI + FES + VR paradigm, which targets gait while minimizing exertional load and aims for durable off-device benefits. Our results therefore align with pwMS preferences for less burdensome electrodes, mobility improvement, and fatigue management, complementing ongoing user-centered design efforts in this population.

Participants demonstrated a robust improvement in walking endurance following the BCI intervention. Adjusted mean increases in 6MWT distance at Post1 (+37.3 m), Post2 (+29.6 m), and Post3 (+21.4 m) were statistically significant, confirming reliable functional gains. Importantly, these changes also exceeded commonly cited minimal clinically important difference (MCID) thresholds for pwMS (19.7 m) (41), indicating patient-relevant benefit. Performance remained above baseline at 6 months, suggesting durability rather than a transient training effect. A modest attenuation from Post1 to Post3 may reflect the underlying neurodegenerative course of MS and the natural decline in gait function over time, yet gains were broadly maintained.

These endurance results are consistent with increased walking speed on the T25FW and improved mobility/balance on the TUG. For T25FW, group mean reductions of approximately 7%–18% approached the pragmatic ≥ 20% benchmark often used to denote clinically meaningful change in MS (with some individuals typically exceeding that threshold even when the group mean does not). TUG times also decreased significantly, a clinically relevant finding given the prevalence of balance and coordination impairments in MS. Although an MS-specific MCID for TUG is not universally established, the magnitude of reduction observed here aligns with values considered meaningful in related neurological populations.

Interpretation of spasticity warrants nuance. MAS scores decreased by 1.3 points, and a ≥ 1-grade drop is commonly judged clinically meaningful. However, MAS is ordinal, has imperfect specificity for neural spasticity versus passive stiffness/contracture, and shows variable inter-rater reliability. Consequently, the convergent reduction in MAS is encouraging but should be interpreted alongside the functional gains rather than as a standalone mechanistic signal.

Patient-reported outcomes improved in parallel. MSIS-29 reductions of 10.18 points exceed commonly cited MCID ranges for the physical domain (8 points) (43), and MFIS reductions of 7.29 points meet or exceed published MCIDs (4 points) (44). The alignment between objective performance (6MWT, T25FW, TUG) and perceived impact of the disease/fatigue (MSIS-29, MFIS) supports a coherent, clinically meaningful effect profile.

Exploratory analyses add context. We found no evidence that gains in 6MWT were directly linked to MS subtype (RRMS, PPMS, SPMS), suggesting that BCI-based gait rehabilitation may be applicable across heterogeneous clinical phenotypes. Notably, improvements tended to be larger among participants with greater baseline disability (higher EDSS). This pattern is plausible and may reflect (i) greater room for improvement (reduced ceiling effects), (ii) prior activity restriction leading to larger detectable gains with structured training, and (iii) the specific action of the BCI protocol—motor-imagery-driven cortical engagement coupled with functional electrical stimulation (FES)—which may be particularly advantageous when corticospinal drive is impaired but partially preserved, strengthening lower-limb musculature without exacerbating fatigue. In practical terms, BCI-FES may offer a viable option for more disabled pwMS who are otherwise limited in their ability to participate in high-effort gait training.

An additional consideration concerns how to contextualize the role of FES. By design, our single-arm feasibility study cannot disentangle the specific contribution of FES alone nor establish the incremental benefit conferred by BCI coupling. Moreover, the effectiveness of FES in MS remains incompletely characterized: most MS–FES investigations evaluate peroneal-nerve stimulators used during everyday ambulation to mitigate foot drop, targeting an orthotic effect (improved gait while the device is active) rather than a therapeutic carryover (benefit persisting after the device is removed). Comparisons with such studies are therefore problematic because (i) post-intervention assessments are often conducted with the device on, (ii) the intervention is continuous and open-ended rather than time-limited, and (iii) off-device outcomes after discontinuation are seldom reported. In contrast, our protocol was restorative rather than assistive: a 6-week, time-limited program that coupled MI-detected neural activity to FES with concurrent visual feedback, with outcomes assessed off device at short-, mid-, and longer-term follow-ups. In our cohort, improvements were maintained for at least 6 months, suggesting a sustained therapeutic carryover. While preliminary, this device-independent approach may support greater autonomy and social participation than strategies that require ongoing daily device use.

Our findings align with prior interventional work by Carrere et al. (22), who evaluated a BCI–FES paradigm in pwMS (*n* = 7; 24 sessions over 8 weeks) and reported clinically meaningful gains in gait speed (T25FW) and walking ability (MSWS-12), accompanied by improved BCI performance and earlier sensorimotor ERD onset (22). We extend this literature by studying a larger cohort (*N* = 26; 24 completers), assessing durability off device up to 6 months, and broadening endpoints to endurance (6MWT), mobility/balance (TUG), spasticity (MAS), and patient-reported outcomes (MSIS-29, MFIS). Using a seated, MI-contingent BCI + FES + VR protocol, we observed durable improvements that corroborate—and extend beyond—the acute effects previously described.

### 4.2 BCI performance

Brain–Computer Interface performance was indexed by motor-imagery classification accuracy (MIAc), which reflects the separability of left- versus right-hand (and ankle) MI patterns—i.e., task-related modulation of lateralized sensorimotor rhythms—relative to chance (50%). Higher MIAc indicates more reliable volitional control of the interface. Three observations merit emphasis. First, although MI-BCI control accuracy was not an inclusion criterion, all enrolled participants achieved above-chance control and received significant contingent feedback. This supports the feasibility of MI-based training in pwMS with moderate-to-severe disability even though some MI BCIs for communication and control are not effective with a minority of users (45–47).

Second, the delivered FES time per session was modest compared with doses commonly reported in assistive FES protocols yet the BCI intervention was associated with clinically meaningful gains. This pattern is consistent with the hypothesis that contingent coupling of stimulation to MI-detected cortical intent increases the efficiency of feedback delivery (i.e., more informative stimulation per unit time) relative to non-neural or continuous stimulation. We caution, however, that cross-study dose comparisons are limited by differences in goals (therapeutic carryover vs. orthotic assistance), paradigms, and reporting practices. Future dose-matched trials should test whether higher MIAc predicts greater functional benefit and whether MI-contingent FES achieves comparable or superior outcomes with reduced stimulation time.

Third, the MIAc for ankle imagery was modestly higher than for hand imagery. A plausible explanation is task salience and attentional allocation: all participants had clinically meaningful lower-limb impairment, which likely increased motivation to engage with ankle MI to obtain contingent stimulation. In a closed-loop paradigm, such reinforcement can enhance separability of the targeted class, yielding higher MIAc.

### 4.3 Limitations

Limitations should be acknowledged. First, this was a single-arm study without a control group or randomization, which precludes causal inference and direct comparison of the BCI intervention with alternative treatments. The functional benefit of the BCI should be explored further, particularly the contribution of the motor-imagery component as the driver of contingent external feedback. Future trials should randomize participants to MI-BCI plus FES versus FES alone (and ideally include a yoked, non-contingent FES control), with blinded outcome assessment and pre-registered primary endpoints, to quantify the incremental effect of closed-loop coupling. Second, the modest sample size reduced statistical power for subgroup analyses (e.g., by MS subtype) and increased the imprecision of effect estimates. Third, BCI performance was summarized primarily by motor-imagery classification accuracy; future work should incorporate EEG-derived biomarkers more tightly linked to mechanism. Candidate markers include the magnitude and laterality of μ−β ERD and the post-movement β rebound (PMBR), geometry-aware separability (Riemannian distance) between MI and rest, sensorimotor network connectivity (e.g., imaginary coherence/wPLI), spatial-pattern stability across sessions, corticomuscular coherence, and error-related potentials. Prospective studies should pre-register such a panel and evaluate test–retest reliability, responsiveness to change, and predictive value for functional outcomes.

Additional work is needed to assess whether MI-BCI with FES could become a practical approach for gait rehabilitation or other goals. Future work could entail: controlled randomized trials including FES-only and sham BCI conditions; other methods to measure brain activity such as fMRI or invasive approaches; additional functional measures; broader physiological assessment involving the EMG, EOG, GSR, HR, and HRV, oxygenation, or other methods; direct electrical or magnetic stimulation of the brain and/or other areas; longer post-intervention assessments; different therapy schedules; multimodal methods to assess confusion, fatigue, or engagement; novel approaches that target specific functional improvements such as spasticity, balance, function, or fatigue; new signal processing and adaptation methods including novel features derived from the EEG, other information, and combinations thereof; broader feedback approaches such as with virtual reality, head-mounted devices or new game-like paradigms; different feedback modalities and paradigms, different methods to support therapy in remote or home environments; more participants with stratification (including by EDSS, medication, or co-morbidities); persons with more severe functional limitations; and other patient groups. Hopefully, the approach presented here and future work from our group and others will support future technologies and systems that help pwMS and other people.

Even if limitations are addressed through future work, widespread practical adoption of any BCI entails broader challenges, risks, and external developments that are difficult for most BCI research groups to control or predict (48). External advances (e.g., novel medications, devices, or therapies), new certifications or regulations, financial hurdles, a public backlash against Artificial Intelligence or BCIs, or the discovery of adverse effects could all reduce the impact of successful BCIs.

Adverse events were monitored at each assessment or therapy session. The safety population included all participants who initiated training (*N* = 26). No adverse events—serious or non-serious—were observed during any training or assessment visit. No participants withdrew due to safety concerns; the two discontinuations were for scheduling/logistical reasons.

In summary, this study provides convergent evidence that BCI-based rehabilitation can produce clinically meaningful improvements in endurance, speed, mobility, and patient-reported outcomes in pwMS, which persist over at least 6 months. These findings support the promise of BCI technology as an accessible, fatigue-sparing complement to SOA gait rehabilitation devices and methods. Future work should include randomized controlled trials against dose-matched conventional therapy; stratification by EDSS and disease activity; incorporation of neurophysiological biomarkers to track plasticity; and evaluation of real-world mobility (e.g., wearables) to strengthen ecological validity and guide patient selection.

## 5 Conclusion

The BCI used in this single-arm study with motor imagery and functional electrical stimulation (MI-BCI + FES) was feasible and safe in pwMS with moderate-to-severe disability. Participants showed clinically meaningful gains in walking endurance, speed, mobility, and patient-reported outcomes, which persisted up to 6 months after the intervention.

## Data Availability

The datasets presented in this article are not readily available because patients’ data need to be treated according to current data protection laws and ethical guidelines. Requests to access the datasets should be directed to guger@gtec.at.

## References

[1] Heesen C Böhm J Reich C Kasper J Goebel M Gold S. Patient perception of bodily functions in multiple sclerosis: gait and visual function are the most valuable. *Mult Scler.* (2008) 14:988–91. 10.1177/1352458508088916 18505775

[2] Cavanaugh J Gappmaier V Dibble L Gappmaier E. Ambulatory activity in individuals with multiple sclerosis. *J Neurol Phys Ther.* (2011) 35:26–33. 10.1097/NPT.0b013e3182097190 21475081

[3] Göksel Karatepe A Kaya T Günaydn R Demirhan A Ce P Gedizlioğlu M. Quality of life in patients with multiple sclerosis: the impact of depression, fatigue, and disability. *Int J Rehabil Res.* (2011) 34:290–8. 10.1097/MRR.0b013e32834ad479 21946317

[4] Larocca N. Impact of walking impairment in multiple sclerosis: perspectives of patients and care partners. *Patient.* (2011) 4:189–201. 10.2165/11591150-000000000-00000 21766914

[5] Petajan JH White AT. Recommendations for physical activity in patients with multiple sclerosis. *Sports Med.* (1999) 27:179–91. 10.2165/00007256-199927030-00004 10222541

[6] Dalgas U Ingemann-Hansen T Stenager E. Physical exercise and ms recommendations. *Int MS J.* (2009) 16:5–11. 19413920

[7] Latimer-Cheung A Pilutti L Hicks A Martin Ginis K Fenuta A MacKibbon K Effects of exercise training on fitness, mobility, fatigue, and health-related quality of life among adults with multiple sclerosis: a systematic review to inform guideline development. *Arch Phys Med Rehabil.* (2013) 94:1800–28.e3. 10.1016/j.apmr.2013.04.020 23669008

[8] Rietberg M Brooks D Uitdehaag B Kwakkel G. Exercise therapy for multiple sclerosis. *Cochrane Database Syst Rev.* (2009) 2005:CD003980. 10.1002/14651858.CD003980.pub2 15674920 PMC6485797

[9] Kuhlmann T Moccia M Coetzee T Cohen J Correale J Graves J Multiple sclerosis progression: time for a new mechanism-driven framework. *Lancet Neurol.* (2022) 22:78–88. 10.1016/S1474-4422(22)00289-7 36410373 PMC10463558

[10] Tavazzi E Cazzoli M Pirastru A Blasi V Rovaris M Bergsland N Neuroplasticity and motor rehabilitation in multiple sclerosis: a systematic review on MRI markers of functional and structural changes. *Front Neurosci.* (2021) 15:707675. 10.3389/fnins.2021.707675 34690670 PMC8526725

[11] Malouin F Jackson PL Richards CL. Towards the integration of mental practice in rehabilitation programs. A critical review. *Front Hum Neurosci.* (2013) 7:576. 10.3389/fnhum.2013.00576 24065903 PMC3776942

[12] Sitaram R Ros T Stoeckel L Haller S Scharnowski F Lewis-Peacock J Closed-loop brain training: the science of neurofeedback. *Nat Rev Neurosci.* (2017) 18:86–100. 10.1038/nrn.2016.164 28003656

[13] Broccard FD Mullen T Chi YM Peterson D Iversen JR Arnold M Closed-loop brain-machine-body interfaces for noninvasive rehabilitation of movement disorders. *Ann Biomed Eng.* (2014) 42:1573–93. 10.1007/s10439-014-1032-6 24833254 PMC4099421

[14] Mayford M Siegelbaum S Kandel E. Synapses and memory storage. *Cold Spring Harb Perspect Biol.* (2012) 4:a005751. 10.1101/cshperspect.a005751 22496389 PMC3367555

[15] Wolpaw J Wolpaw EW. *Brain–Computer InterfacesPrinciples and Practice.* Oxford: Oxford University Press (2012). 10.1093/acprof:oso/9780195388855.001.0001

[16] Jackson PL Lafleur MF Malouin F Richards C Doyon J. Potential role of mental practice using motor imagery in neurologic rehabilitation. *Arch Phys Med Rehabil.* (2001) 82:1133–41. 10.1053/apmr.2001.24286 11494195

[17] García Carrasco D Aboitiz Cantalapiedra J. Effectiveness of motor imagery or mental practice in functional recovery after stroke: a systematic review. *Neurologia.* (2016) 31:43–52. 10.1016/j.nrl.2013.02.003 23601759

[18] Simmons L Sharma N Baron JC Pomeroy VM. Motor imagery to enhance recovery after subcortical stroke: Who might benefit, daily dose, and potential effects. *Neurorehabil Neural Repair.* (2008) 22:458–67. 10.1177/1545968308315597 18780881

[19] Guillot A Di Rienzo F Macintyre T Moran A Collet C. Imagining is not doing but involves specific motor commands: a review of experimental data related to motor inhibition. *Front Hum Neurosci.* (2012) 6:247. 10.3389/fnhum.2012.00247 22973214 PMC3433680

[20] Gil-Bermejo-Bernardez-Zerpa A Moral-Munoz J Lucena-Anton D Luque-Moreno C. Effectiveness of motor imagery on motor recovery in patients with multiple sclerosis: systematic review. *Int J Environ Res Public Health.* (2021) 18:498. 10.3390/ijerph18020498 33435410 PMC7827037

[21] Hanson M Concialdi M. Motor imagery in multiple sclerosis: exploring applications in therapeutic treatment. *J Neurophysiol.* (2019) 121:347–9. 10.1152/jn.00291.2018 30207860

[22] Carrere L Taborda M Ballario C Tabernig C. Effects of brain-computer interface with functional electrical stimulation for gait rehabilitation in multiple sclerosis patients: preliminary findings in gait speed and event-related desynchronization onset latency. *J Neural Eng.* (2021) 18: 10.1088/1741-2552/ac39b8 34781272

[23] Liberson W Holmquest H Scot D Dow M. Functional electrotherapy: stimulation of the peroneal nerve synchronized with the swing phase of the gait of hemiplegic patients. *Arch Phys Med Rehabil.* (1961) 42: 101–5. 13761879

[24] Hong Z Sui M Zhuang Z Liu H Zheng X Cai C Effectiveness of neuromuscular electrical stimulation on lower limbs of patients with hemiplegia after chronic stroke: a systematic review. *Arch Phys Med Rehabil.* (2018) 99:1011–22.e1. 10.1016/j.apmr.2017.12.019. 29357280

[25] Esnouf JJE Taylor PN Mann GE Barrett CL. Impact on activities of daily living using a functional electrical stimulation device to improve dropped foot in people with multiple sclerosis, measured by the Canadian occupational performance measure. *Multiple Sclerosis.* (2020) 16:1141–7. 10.1177/1352458510366013 20601398

[26] Mayer L Warring T Agrella S Rogers H Fox E. Effects of functional electrical stimulation on gait function and quality of life for people with multiple sclerosis taking dalfampridine. *Int J MS Care.* (2015) 17:35–41. 10.7224/1537-2073.2013-033 25741225 PMC4338641

[27] Downing A Van Ryn D Fecko A Aiken C McGowan S Sawers S Effect of a 2-week trial of functional electrical stimulation on gait function and quality of life in people with multiple sclerosis. *Int J MS Care.* (2014) 16:146–52. 10.7224/1537-2073.2013-032 25337057 PMC4204375

[28] Renfrew L Paul L McFadyen A Rafferty D Moseley O Lord A The clinical- and cost-effectiveness of functional electrical stimulation and ankle-foot orthoses for foot drop in Multiple Sclerosis: a multicentre randomized trial. *Clin Rehabil.* (2019) 33:1150–62. 10.1177/0269215519842254 30974955

[29] Miller L McFadyen A Lord A Hunter R Paul L Rafferty D Functional electrical stimulation for foot drop in multiple sclerosis: a systematic review and meta-analysis of the effect on gait speed. *Arch Phys Med Rehabil.* (2017) 98:1435–52. 10.1016/j.apmr.2016.12.007 28088382

[30] Gulcan K Guclu-Gunduz A Yasar E Ar U Sucullu Karadag Y Saygili F. The effects of augmented and virtual reality gait training on balance and gait in patients with Parkinson’s disease. *Acta Neurol Belg.* (2023) 123:1917–25. 10.1007/s13760-022-02147-0 36443623 PMC9707084

[31] Feng H Li C Liu J Wang L Ma J Li G Virtual reality rehabilitation versus conventional physical therapy for improving balance and gait in Parkinson’s disease patients: a randomized controlled trial. *Med Sci Monit.* (2019) 25:4186–92. 10.12659/MSM.916455 31165721 PMC6563647

[32] Cervera M Soekadar S Ushiba J Millán J Liu M Birbaumer N Brain-computer interfaces for post-stroke motor rehabilitation: a meta-analysis. *Ann Clin Transl Neurol.* (2018) 5:651–63. 10.1002/acn3.544 29761128 PMC5945970

[33] Tekeoglu Tosun A Ipek Y Razak Ozdincler A Saip S. The efficiency of mirror therapy on drop foot in multiple sclerosis patients. *Acta Neurol Scand.* (2021) 143:545–53. 10.1111/ane.13385 33270229

[34] Ryan D Fullen B Rio E Segurado R Stokes D O’Sullivan C. Effect of action observation therapy in the rehabilitation of neurologic and musculoskeletal conditions: a systematic review. *Arch Rehabil Res Clin Transl.* (2021) 3:100106. 10.1016/j.arrct.2021.100106 33778479 PMC7984987

[35] Chow S-C Wang H Shao J. *Sample Size Calculations in Clinical Research.* Boca Raton, FL: Chapman and Hall (2007). 10.1201/9781584889830

[36] Baert I Freeman J Smedal T Dalgas U Romberg A Kalron A Responsiveness and clinically meaningful improvement, according to disability level, of five walking measures after rehabilitation in multiple sclerosis: a European multicenter study. *Neurorehabil Neural Repair.* (2014) 28:621–31. 10.1177/1545968314521010 24503204

[37] Meseguer-Henarejos AB Sáchez-Meca J López-Pina JA Carles-Hernández R. Inter-and intra-rater reliability of the modified ashworth scale: a systematic review and meta-analysis. *Eur J Phys Rehabil Med.* (2018) 54:576–90. 10.23736/S1973-9087.17.04796-7 28901119

[38] Sebastián-Romagosa M Cho W Ortner R Sieghartsleitner S Von Oertzen T Kamada K Brain-computer interface treatment for gait rehabilitation in stroke patients. *Front Neurosci.* (2023) 17:1256077. 10.3389/fnins.2023.1256077 37920297 PMC10618349

[39] Sebastián-Romagosa M Cho W Ortner R Murovec N Von Oertzen T Kamada K Brain computer interface treatment for motor rehabilitation of upper extremity of stroke patients-A feasibility study. *Front Neurosci.* (2020) 14:591435. 10.3389/fnins.2020.591435 33192277 PMC7640937

[40] Ortner R Irimia D Scharinger J Guger C. A motor imagery based brain-computer interface for stroke rehabilitation. *Stud Health Technol Inform.* (2012) 181:319–23. 22954880

[41] Oosterveer D van den Berg C Volker G Wouda N Terluin B Hoitsma E. Determining the minimal important change of the 6-minute walking test in multiple sclerosis patients using a predictive modelling anchor-based method. *Mult Scler Relat Disord.* (2022) 57:103438. 10.1016/j.msard.2021.103438 34871859

[42] Russo J Mahoney T Kokorin K Reynolds A Lin C John S Towards developing brain-computer interfaces for people with multiple sclerosis. *PLoS One.* (2025) 20:e0319811. 10.1371/journal.pone.0319811 40100843 PMC11918325

[43] Costelloe L O’Rourke K Kearney H McGuigan C Gribbin L Duggan M The patient knows best: significant change in the physical component of the multiple sclerosis Impact Scale (MSIS-29 physical). *J Neurol Neurosurg Psychiatry.* (2007) 78:841–4. 10.1136/jnnp.2006.105759 17332049 PMC2117755

[44] Rooney S McFadyen D Wood D Moffat D Paul P. Minimally important difference of the fatigue severity scale and modified fatigue impact scale in people with multiple sclerosis. *Mult Scler Relat Disord.* (2019) 35:158–63. 10.1016/j.msard.2019.07.028 31400557

[45] Allison BZ Neuper C. *Could Anyone Use a BCI?.* Berlin: Springer (2010). 10.1007/978-1-84996-272-8_3

[46] Vidaurre C Blankertz B. Towards a cure for BCI illiteracy. *Brain Topogr.* (2010) 23:194–8. 10.1007/s10548-009-0121-6 19946737 PMC2874052

[47] Thompson M. Critiquing the concept of BCI Illiteracy. *Sci Eng Ethics.* (2019) 25:1217–33. 10.1007/s11948-018-0061-1 30117107

[48] Schalk G Brunner P Allison B Soekadar S Guan C Dension T Translation of neurotechnologies. *Nat Rev Bioeng.* (2024) 8:637–52. 10.1038/s44222-024-00185-2

